# Resveratrol Based Oral Nutritional Supplement Produces Long-Term Beneficial Effects on Structure and Visual Function in Human Patients

**DOI:** 10.3390/nu6104404

**Published:** 2014-10-17

**Authors:** Stuart Richer, Shana Patel, Shivani Sockanathan, Lawrence J. Ulanski, Luke Miller, Carla Podella

**Affiliations:** 1 Eye Clinic 112e, Captain James A Lovell Federal Health Care Center, 3001 Green Bay Rd., North Chicago, IL 60064, USA; E-Mails: larry.ulanski@gmail.com (L.J.U.); cjpstella63@gmail.com (C.P.); 2 University of Illinois Eye and Ear Infirmary, University of Illinois at Chicago, Chicago, IL 60612, USA; E-Mail: Lulanski@uic.edu; 3 Chicago Medical School, Rosalind Franklin University of Medicine and Science, 3333 Green Bay Rd., North Chicago, IL 60064, USA; E-Mails: shana.patel@my.rfums.org (S.P.); shivani.sockanathan@my.rfums.org (S.S.); luke.miller@my.rfums.org (L.M.)

**Keywords:** epigenetics, visual structure and function, resveratrol, Longevinex^®^, age-related macular degeneration, ophthalmology

## Abstract

Background: Longevinex^®^ (L/RV) is a low dose hormetic over-the-counter (OTC) oral resveratrol (RV) based matrix of red wine solids, vitamin D_3_ and inositol hexaphosphate (IP6) with established bioavailability, safety, and short-term efficacy against the earliest signs of human atherosclerosis, murine cardiac reperfusion injury, clinical retinal neovascularization, and stem cell survival. We previously reported our short-term findings for dry and wet age-related macular degeneration (AMD) patients. Today we report long term (two to three year) clinical efficacy. Methods: We treated three patients including a patient with an AMD treatment resistant variant (polypoidal retinal vasculature disease). We evaluated two clinical measures of ocular structure (fundus autofluorescent imaging and spectral domain optical coherence extended depth choroidal imaging) and qualitatively appraised changes in macular pigment volume. We further evaluated three clinical measures of visual function (Snellen visual acuity, contrast sensitivity, and glare recovery to a cone photo-stress stimulus). Results: We observed broad bilateral improvements in ocular structure and function over a long time period, opposite to what might be expected due to aging and the natural progression of the patient’s pathophysiology. No side effects were observed. Conclusions: These three cases demonstrate that application of epigenetics has long-term efficacy against AMD retinal disease, when the retinal specialist has exhausted other therapeutic modalities.

## 1. Introduction

There is emerging interest in applying epigenetics to the field of ophthalmology, ocular health status, function, and disease(s). Epigenetic modification using small molecular weight nutrients has been discussed in cancer and heart disease, suggesting a potential for cardio-protection against ischemia-reperfusion injuries [1]. However, there remains a dearth of publications applying epigenetics to clinical ophthalmology. There are fewer than a hundred accumulated published articles on PubMed concerning epigenetic regulatory mechanisms and ocular development, health, and diseases.

Epigenetics involves modifications of genetic material that does not impact DNA nucleotide sequences. The complex epigenome results in gene expression and/or gene silencing. While classical genetics concerns itself with inherited mutations (*i*.*e*., nucleotide substitutions, omissions), epigenetics involves the variable protein expression that facilitates cellular adaptability, enhancing stem cell survival, and tissue regeneration as manifested through the three specific mechanisms:

(1) DNA methylation, the addition of a methyl group (M) to the DNA base cytosine (C) from folate (B_9_) and B_12_, is most active during embryogenesis and early childhood.
(2) Histone modification include protein spools that control gene expression by winding or unwinding DNA strands around histone bodies by such natural/dietary histone deacetylase (HDAC) inhibitors as sulforaphane in cruciferous vegetables, diallyl disulfide in garlic, nigella sativa in black cumin seed oil, and resveratrol (RV) in red wine.
(3) MicroRNA, short 22 base pair strands of RNA that mesh with messenger RNA, silences a segment of genes during translation. Therefore, microRNAs block the protein-making ability of gene(s). MicroRNA is called the “guiding hand of the human genome” [2].

Significantly, these processes are controlled by environmental cues such as temperature, radiation, oxygen deprivation, food or food deprivation, (the latter doubling the healthspan and lifespan of lab animals as first demonstrated by Clive McCay in the 1930s) [3], as well as provision of small molecular weight molecules in nutrients, such as allicin (garlic) and RV (red wine).

Epigenetic mechanisms have great potential to improve the status and function of ocular tissue. Age-related macular degeneration (AMD) is one of the leading causes of progressive and irreversible vision loss in Western societies. AMD can progress typically from dry (non-exudative) to wet (exudative) form, where the wet form is often visually catastrophic. AMD is distinguished from genetically inherited forms of retinal disease such as Leber’s and Stargardt’s hereditary retinal dystrophies. This multifactorial aging disorder involves a combination of aging, polygenetic susceptibility genes, and myriad environmental factors, such as smoking and obesity. Epigenetic modifications of the genome modulate this phenotypical variability [4].

RV, a non-flavonoid polyphenol phytoalexin toxin, is produced in response to infection in plants, such as *Vitis vinifera* (a grape) when cultivated in stressful conditions, such as cold or lack of light (*i*.*e*., northern altitudes) [5]. This small molecule (molecular weight 228 Daltons) enters all cells, does not need to be nanosized, and acts as a dietary histone deacetylase inhibitor, altering the winding and modulating protective pathways against oxidative stress, DNA damage, excitotoxicity, apoptosis, and inflammation. The activity of RV has been linked to cell-surface receptors, membrane signaling pathways, intracellular signal-transduction machinery, nuclear receptors, gene transcription, and metabolic pathways [6]. RV has germane ophthalmologic biological actions, which include anti-angiogenesis activity and choroidal vasorelaxation [7]. Numerous animal studies have demonstrated that this polyphenol holds promise against numerous age-associated diseases including cancer, diabetes, Alzheimer, and cardiovascular and pulmonary diseases [5].

Short-term anti-neovascular effects of Longevinex (L/RV), a resveratrol-containing supplement, on AMD patients have been published [8], and further mechanistic validation of RVs’ suppression of VEGF by human RPE cells has recently been published [9]. We present our clinical experience in the clinic over a multi-year period.

## 2. Experimental Section

### 2.1. Selecting an Oral OTC-RV AMD Supplement with Established Bioavailability and Short-Term Efficacy

There is a false concern over RVs’ short half-life (minutes) and its lack of bioavailability. Rapid conjugation with glucuronate and sulfate during hepatic detoxification dramatically extending its half-life to 9 h rather than minutes [10]. Generic RV can be photo-isomerized from *trans* to *cis* RV by exposure to ultraviolet radiation. For these reasons we selected a commercially available brand of resveratrol that had been stabilized via microencapsulation and tested and shown to work superiorly to plain resveratrol and has undergone toxicity testing [11]. L/RV, an OTC oral RV-based supplement, contains microencapsulated/microionized RV and a blend of red wine polyphenols, primarily quercetin, along with vitamin D_3_, and inositol hexaphosphate (IP6). The process of microencapsulation and miconization of *trans*-RV enhances RV stability to light, heat, and oxygen, while micronization drastically increases blood concentration, lifespan, and epigenetic biological potency.

Studies have shown that quercetin enhances the immediate bioavailability of RV (RV makes more passes through the liver before complete metabolization). Vitamin D_3_ controls a variety of genes involved in innate immunity, inflammation, and vascular calcification and has recently emerged as an important nutrient (prohormone) against AMD [12,13]. IP6 (inositol hexaphosphate) from rice bran is nature’s divalent mineral binder (*i*.*e*., iron and copper) and replaces the need for phlebotomy or chelation, in diseases of iron mismanagement [14]. Multiple polyphenols, in general, and L/RV in particular, exhibit synergistic rather than just additive biological and epigenetic effects [15].

The published synergistic actions of L/RV itself, and/or reference to a component within L/RV, are found at Longevinex^®^ [16], and extend well beyond RV to include:

(1) ↓ Inflammation (COX-2, CRP, TNF)
(2) ↓ HIF-1 and VEGF genes (microRNA 21, 20b, 539)
(3) ↑ Nrf2 endogenous antioxidants (glutathione, SOD, catalase) via the Nrf2 gene.
(4) ↓ Blood clotting (platelet stickiness)
(5) ↑ Vasodilation (nitric oxide)
(6) ↑ Metal chelation (copper)
(7) ↓ Oxidation, peroxidation (mega-dose increases oxidation)
(8) ↓ Cell adhesion (platelets, microbes, tumor)
(9) ↓ Calcification (*i*.*e*., Bruch’s membrane)
(10) ↓ Bacteria, viruses, fungi
(11) ↑ Kruppel-like factor 4 (KLF4) activities via RV and vitamin D_3_, responsible for anti-VEGF therapy failure

Short-term L/RV anti-VEGF type improvements even in octogenarians with ocular pathology, was recently documented at our medical center [8]. L/RV acts as an anti-oxidant to reduce oxidative stress, and our group was also the first to show minimized lipofuscin epoxidation in an octogenarian AMD patient presumably by down-regulation of reactive oxygen species, sparing aging pigment epithelial cells from DNA damage and premature apoptosis, as well as promoting stem cell survival [17].

L/RV activates miRNA genes in murine models nine-fold greater than RV alone [18]. Through such effects, L/RV may outperform anti-VEGF monoclonal antibodies. This can be accomplished because L/RV addresses the known reason why anti-VEGF drugs fail (macrophage polarization via KLF4) and L/RV has superior down-regulation of miRNA 20b expression 1366 times [18] compared to plain RV. The miRNA 20b fragment controls the hypoxia-inducing factor-1 (HIF1) and the VEGF gene, decreasing VEGF to prevent choroidal neovascularization and angiogenesis in as little as one week [8]. This mechanism prevents the conversion from dry to wet AMD.

We have also clinically shown, using spectral domain OCT imaging, that L/RV targets and promotes RPE stem cells to regenerate within the living human eye of another octogenarian (88-year-old) AMD patient, over a 20-week period [19]. Unfortunately, large scale L/RV human clinical trials are lacking. However, a clinical trial in Japan, has demonstrated cardiovascular benefits of L/RV through improvements in endothelial function of adults with metabolic syndrome receiving standard treatment [20].

### 2.2. Dosage and Clinical Safety Considerations

RV characteristically exhibits a U or J shaped risk curve with hormetic low dose benefits and negative effects at high doses [1]. In rodents it is cardio-protective between 175 and 350 mg human equivalent dose (HED), and cytotoxic at 10-times higher doses between 1750 and 3500 mg HED, and a dose of 3500 mg RV was universally lethal to rat hearts [1]. A Glaxo Smith Kline phase 2 human clinical trial of RV in multiple myeloma patients was suspended in 2010 because several patients developed kidney failure at 5000 mg.

In contradistinction, L/RV has been shown to exhibit a biologically unique L-shaped risk curve and unparalleled margin of safety at high doses up to 2800 mg HED. This margin of safety is clearly superior to RV alone. L/RV passed toxicity testing, meeting the New Dietary Ingredient guidelines described by the FDA [11,21]. This dietary supplement exhibits good safety and the recommended dosage (one capsule per day) and has not produced major side effects among non-anemic subjects over a ten-year period of commercial use [16].

Veteran patients at the James A Lovell Federal Health Care Center, for whom other therapeutic measures had been exhausted (ARED and AREDS II supplements, anti-VEGF treatments, *etc*.), were prescribed one capsule containing low dose nutrients (100 mg *trans* RV) daily and followed clinically. L/RV was compassionately provided when no other options were available beyond the Vision Impairment Service Teams’ low vision and blind-rehabilitation services.

### 2.3. Consent/IRB

An Investigational New Drug (IND) submission is not required to use a marketed product as a part of medical practice for individual patients. However, supervision was requested and approved by the Chief of Staff and IRB (Hines DVA, Chicago, IL, USA).

### 2.4. Participants

We compiled data from three male AMD patients who were on the L/RV matrix for multi-year periods using an enhanced clinical visual function protocol developed at our medical center [22].

1. Patient 1, a 64-year-old Caucasian glaucoma suspect with photophobia, atrophic AMD (worse right eye), and diabetes with declining vision function right eye, has been on L/RV for 2.5 years and is maintaining visual function.
2. Patient 2, an 89-year-old Caucasian with chronic kidney disease and cataracts, has been on L/RV for 3 years maintaining his visual function requirements to retain his driver’s license.
3. Patient 3, a 67-year old Caucasian with bilateral Polypoidal Choroidal Vasculopathy (PCV), a treatment resistant AMD variant, worse right eye. He also has a history of central serous retinopathy above the right optic nerve and a left retinal central foveal photoreceptor integrity line defect and impaired color vision. Improved retinal/choroid structure was observed.

### 2.5. Methodology

Retina/choroid structure modalities:

1. Retinal Pigment Epithelium (RPE) Fundus Auto Fluorescence (FAF) images were obtained with Optos^®^ 200Tx, (Optos PLC, Dunfermline, UK), a wide-field camera that images the ocular fundus using specialized laser light. It captures a 200-degree field in one image, as compared to standard cameras that image only 50 degrees, allowing for extended evaluation. FAF images were obtained in order to identify lipofuscin granule accumulation in the lysosomal compartment of RPE cells Lipofuscin, is a mixture of autofluorescent pigments that accumulates in the RPE as a byproduct from incomplete degradation of photoreceptor outer segment, and is associated with the disease progression in AMD and retinal disease.
2. Retinal Spectral Domain Optical Coherence Tomographic (SD OCT) images were obtained with an RTVue^®^ instrument (OptoVue, Freemont, CA, USA). These images depict precisely aligned high-resolution *in vivo* histologic sagittal retinal cross sections as well as “extended-depth” choroidal vasculature cross-section images. The OCT highlights retinal alterations in morphology, structure and reflectivity, facilitating baseline and serial clinical evaluation of individual retinal layers. Using “extended depth SD-OCT” the choroid is visible. Choroid thickness decreases with age and even faster in AMD and glaucoma patients, suggestive of diminished choroidal perfusion at least partially responsible for a decline in visual function.
3. Visualized macular pigment volume imaged by ARIS^®^ (Automated Retinal Imaging System) (Visual Pathways, Inc., Prescott, AZ, USA) that also depicts retinal layers by spectral separation. We employ the spectral (visible/IR) separation images for AMD patients because, compared to traditional fundus photographs, there is high sensitivity in identifying intra-retinal pathology (retinal drusen), the critical underlying blood supply underneath the retina (*i*.*e*., the choroidal network that becomes less dense in AMD), as well as MPOD typically diminished in AMD. Traditional colored fundus photographs are also derived through wavelength recombination.

Visual function modalities:

1. Visual Acuity (VA—high contrast, high spatial frequency): The clinical best-refracted Snellen acuity was taken in a semi-darkened room using a digital Smart Systems^®^ projection system (M and S Technologies, Skokie, IL, USA).
2. Contrast Sensitivity (CSF—all contrasts, all spatial frequencies): The contrast sensitivity function (CSF), a measure of how an eye sees large objects (low spatial frequencies at 1.5 and 3 cycles/degree) and small objects such as Snellen letters (higher spatial frequencies, *i*.*e*., 18 cycles/degree)—*x* axis, at differing contrasts—*y* axis. The area under the curve of the resulting CSF at five spatial frequencies was measured with the validated instrument (Functional Vision Analyzer^®^ (Stereo Optical, Chicago, IL, USA) with best refraction at each visit.
3. Glare Recovery (GR—in seconds): Photo-stress cone glare recovery in seconds to a bright flash that induces retinal-RPE dysfunction was measured with a validated clinical Macular Disease Detection MDD-2^®^ macular adaptometer (Health Research Sciences, Lighthouse Point, FL, USA).

## 3. Results

Cases 1–3 present patients afflicted with AMD that have taken L/RV over a multi-year period. All other curative measures had been exhausted for these patients, or the patient was either refused intra-vitreal injections, treatment resistant, or classified as non-candidates by conventional retinal ophthalmologic evaluation.

**Figure 1:**
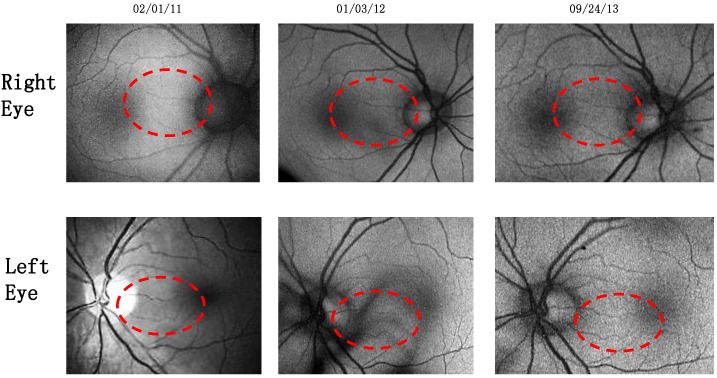
Wide field OPTOS^®^ FAF images of lipofuscin within the neurosensory retina of a 64-year-old male patient with AMD and diabetes type 2. These images are original, untouched, and unaltered. Over time, there is a decrease in autofluorescence in both retinas signifying a decrease in lipofuscin accumulation. Image (*i.e.*, below, center, middle) includes the patient’s eyelashes (artifact).

**Figure 2:**
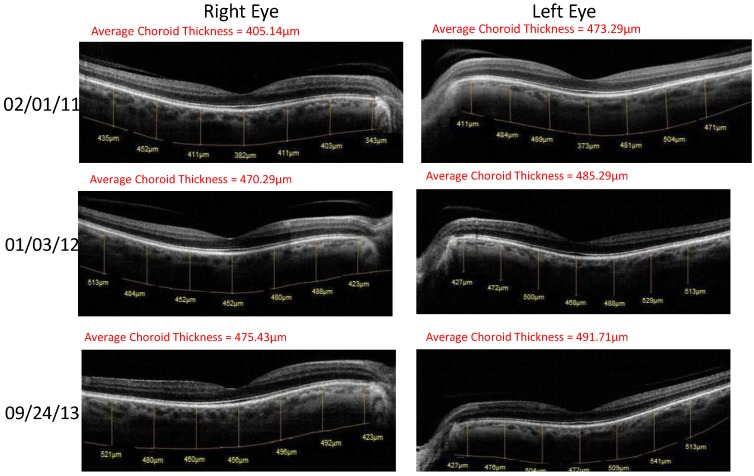
SDOCT “Extended Depth Imaging” of chorodal thickness (microns) of a 64-year-old male patient with AMD and diabetes type 2. Over time, approximately 2.5 years, there is an increase in choroid thickness in both retinas denoting better choroidal perfusion, which is opposite of the expected age-related trend.

**Figure 3:**
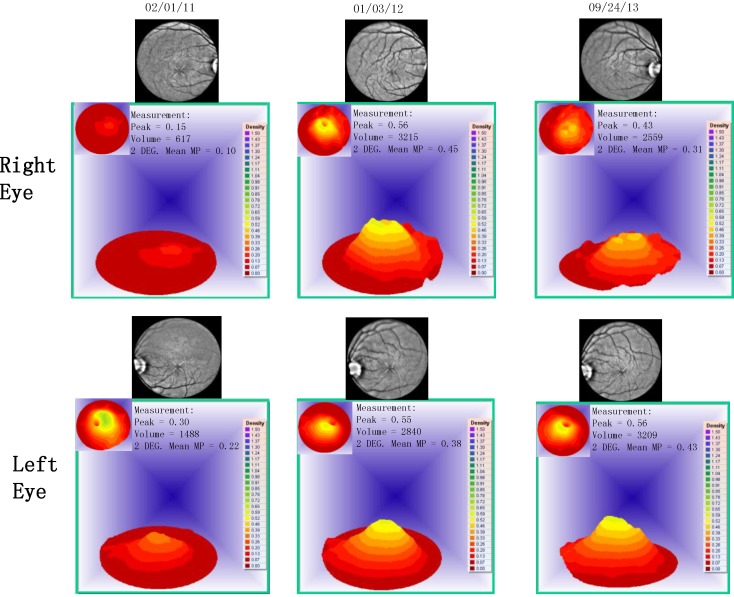
Visualized macular pigment volume of a 64-year-old male patient with AMD and diabetes type 2. With time, approximately 2.5 years, there is bilateral increased macular pigment with no change in reported dietary or supplemental carotenoid/omega-3 intake, an unusual clinical finding.

**Figure 4:**
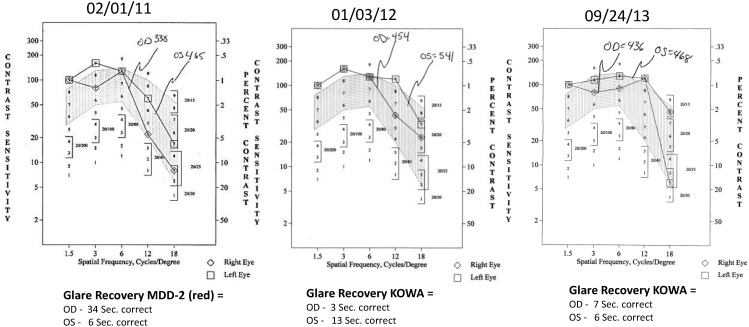
Contrast Sensitivity and Glare Recovery of a 64-year-old male patient with AMD and diabetes type 2. With time, approximately 2.5 years, comprehensive measures of visual function, such as contrast sensitivity, improve in both eyes while glare recovery to a photo stress stimulus improves in his left eye. This is opposite of the expected AMD related decline. Baseline Snellen visual acuity was 20/25 R, 20/20 L and further improved in the right eye to 20/20 by the second visit within one year.

Case 1: A 64-year-old patient with atrophic AMD and diabetes type 2 taking L/RV for 2.5 years. We observed diminishment of retinal lipofuscin, enhanced choroidal thickness (perfusion), qualitatively increased visualized macular pigment volume, CSF, and shortened GR time. Figure 1 depicts decreased RPE FAF over time reflecting lipofuscin resolution in both retinas. Figure 2 shows choroidal thickening, opposite of an expected age-related decline. Figure 3 depicts dramatic improvements in macular pigment volume with no change in carotenoid/omega-3 supplementation. These factors denote improved ocular structure. Figure 4 depicts better CSF and GR over time. Cardiovascular and serum parameters were also reviewed. We observed improvements in HbA1C, body mass index (BMI), blood pressure (BP), pulse, and pulse pressure (Table 1). The following measurements were taken during patient visits before L/RV use (*n =* 4 from 9/29/10 to 12/9/10) and patient visits after L/RV use (*n =* 16 from 2/25/11 to 6/12/13); A1C levels decreased by 0.36 mg%; BMI decreased by 1.87; systolic BP decreased by 13.37 mmHg and diastolic BP decreased by 2.69 mmHg; pulse decreased by 9.87 beats/min; pulse pressure decreased by 10.69 mmHg. The patient has been pleased with both his enhanced vision and improved cardiovascular health.

**Table 1:** Cardiovascular and serum parameters of a 64-year-old male patient with AMD and diabetes type 2. There are broad improvements over time in his HBA1c, BMI (body mass index), systolic, diastolic, pulse and pulse-pressure. Measurements were taken from his medical records months before starting L/RV and months following initiation of epigenetic therapy (see text for details).

Parameter	Average (SD) before Longevinex	Average (SD) While on Longevinex
A1C	6.43 (0.26)	6.07 (0.35)
BMI	35.75 (0.96)	33.88 (0.89)
BP-Systolic	129.50 (4.04)	116.13 (10.19)
BP-Diastolic	76.50 (8.02)	73.81 (6.28)
Pulse	96.50 (9.33)	86.63 (7.23)
Pulse-Pressure	53.00 (7.53)	42.31 (10.52)
RDW	12.42 (1.02)	13.95 (0.49)

**Figure 5:**
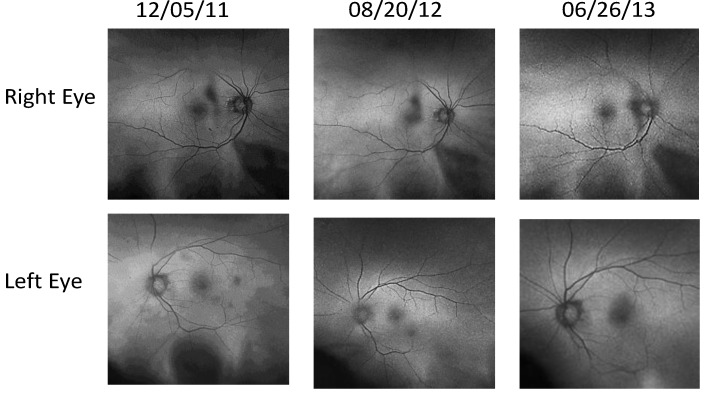
Wide field OPTOS FAF images of lipofuscin within the neurosensory retina of an 89-year-old male patient with AMD. Images of lipofuscin within broad geographic section of the retina is shown to diminish in intensity over time, especially within his left retina.

**Figure 6:**
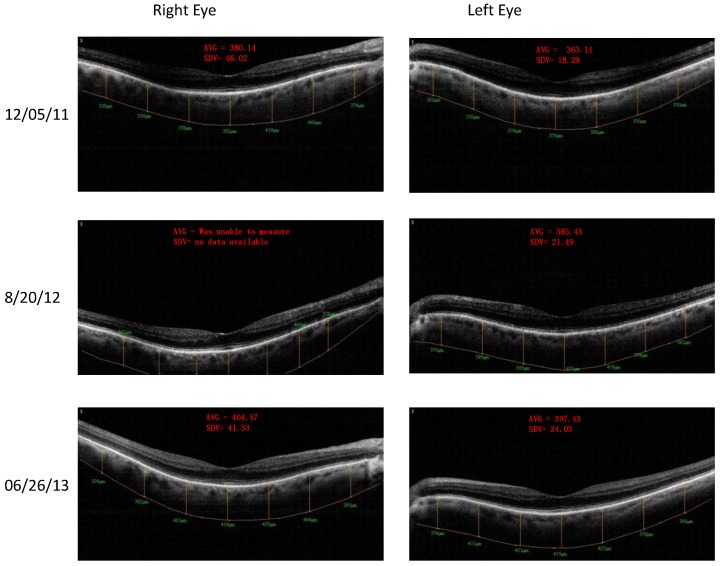
SDOCT “Extended Depth Imaging” of Choroidal Thickness (microns) of an 89-year-old male patient with AMD. With time, approximately 2.5 years, the difference from baseline is +24.4 um of additional right eye choroidal thickening, while the difference from baseline is even greater +34.3 um in the left eye. This represents additional choroidal thickening, well above the 5.0-micron axial resolution of the OptoVue RTVue^®^ SD OCT. Better clinical perfusion of the posterior retina is surmised.

**Figure 7:**
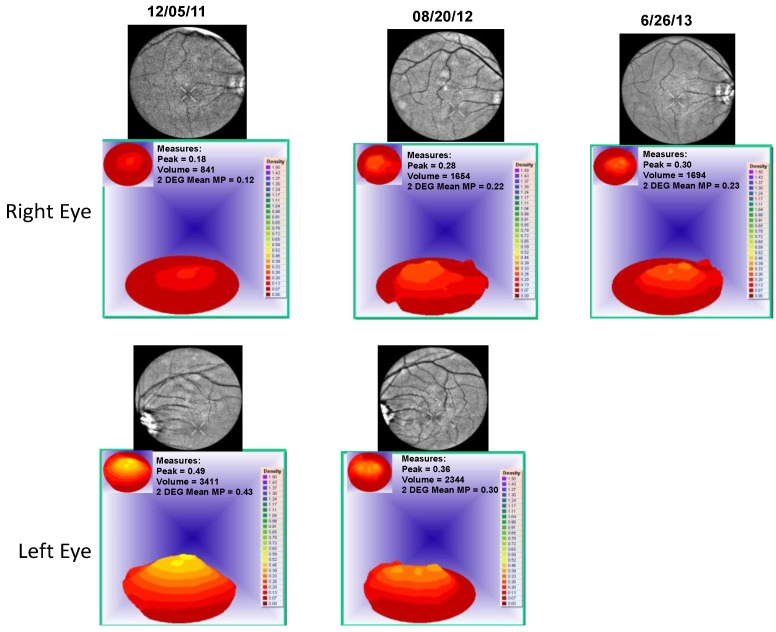
Visualized macular pigment volume of an 89-year-old male patient with AMD. No change in macular pigment over time in both retinas is seen. The patient could not focus on the marker to take 500 nm specular image of left retina on 26 June 2013.

**Figure 8:**
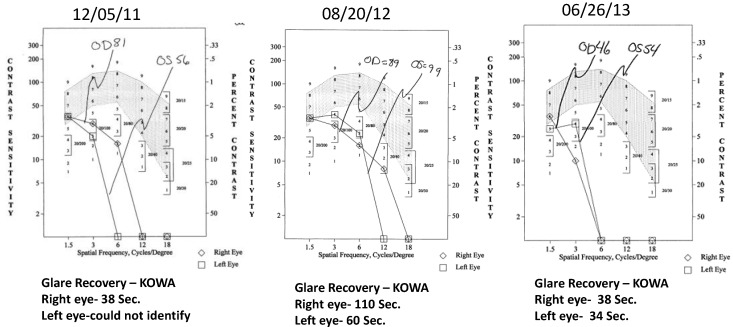
Contrast Sensitivity and Glare Recovery of an 89-year-old male patient with AMD. The contrast sensitivity and glare recovery function right eye are stable, while his left eye has progressive dramatically improved glare recovery (in seconds). Both eyes have an abnormally prolonged glare recovery time that improved but was still abnormal. Expected is 10 to 12 s, in individuals without retinal pathology. This is opposite of the anticipated aging and pathophysiological decline. Baseline Snellen visual acuity was 20/40 in each eye and remained unchanged over 2.5 years allowing the octogenarian to maintain an unrestricted driver’s license.

Case 2: An 89-year-old Caucasian male with atrophic AMD who has been on L/RV for 2.5 years. We similarly observed a decrease in lipofuscin accumulation (Figure 5), an increase in choroidal thickness (Figure 6), stabilization of visualized macular pigment volume (Figure 7), stabilization of GR right eye, better GR left eye, and better CSF (Figure 8). The patient continues to maintain an unrestricted driver’s license, despite his age and expected pathologic decline.

**Figure 9:**
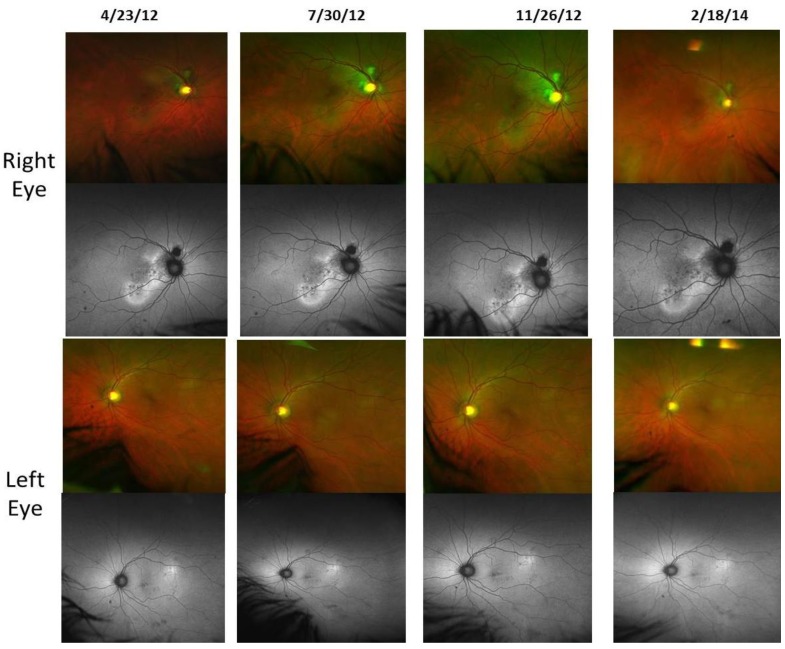
Wide Field OPTOS^®^ FAF Images of Lipofuscin within the Neurosensory Retina of a 67-year-old male with Polypoidal Choroidal Vasculopathy (PCV), a “treatment resistant” AMD variant. No fluorescein angiography was possible due to a previous allergic reaction to iodine. There is a decrease in auto fluorescence evident in his right retina by Month 22.

**Figure 10:**
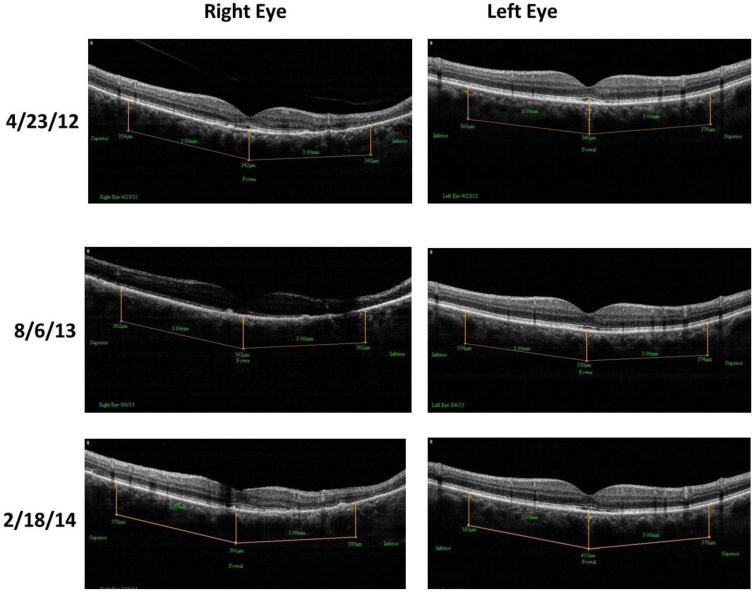
SDOCT “Extended Depth Imaging” of Choroidal Thickness (microns) of a 67-year-old male patient with PCV, an AMD treatment resistant variant. Choroidal thickness (in microns) increased 12% difference right eye and 7% difference left eye by 18 months. This represents additional choroidal thickening, well above the 5.0-micron axial resolution of the OptoVue RTVue^®^ SD OCT. Better clinical perfusion of the posterior retina is presumed.

**Figure 11:**
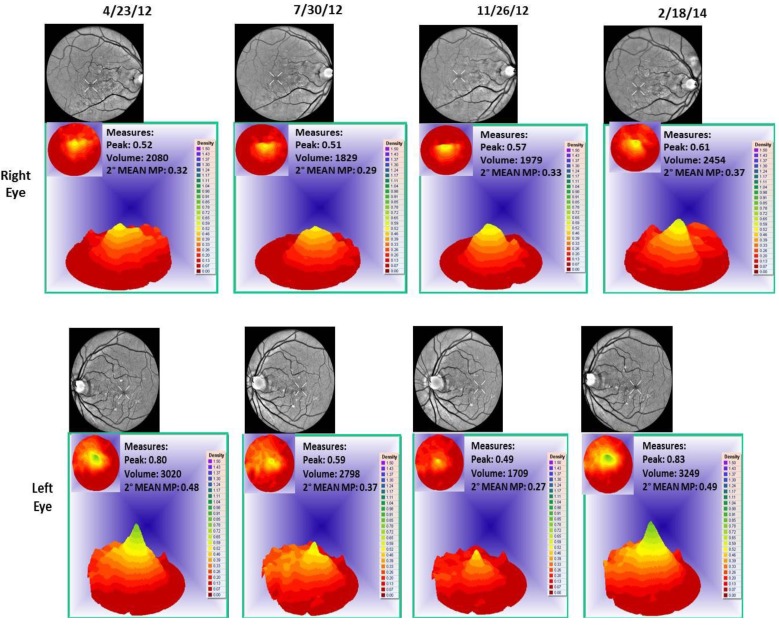
Visualized macular pigment volume, of our 67-year-old male patient with PCV. With time, there is a continual increase in macular pigment volume in the right macula. There was no change in reported dietary or supplemental carotenoid/omega-3 intake. This is an unusual clinical finding, denoting improved cellular health.

**Figure 12:**
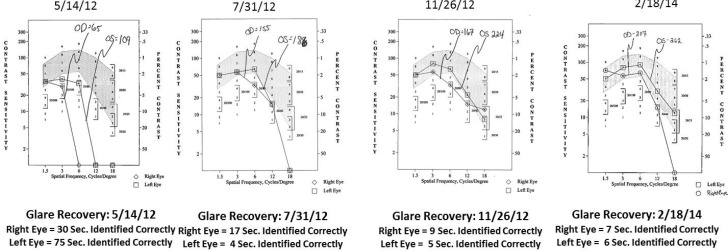
Contrast Sensitivity and Glare Recovery of a 67-year-old male patient with PCV, an AMD variant, and few clinical options. This PCV patient had deteriorated CSF (R 65 AUC, L 109 AUC) at baseline. Normal values are between 200 and 300 AUC. By the second visit, 10 weeks of taking L/RV, the CSF had substantially improved (155 R, 186 L) with continual improvement at the third visit and normalization of each eye by 22 months. The glare recovery quickened dramatically from an abnormal prolonged 30 s right eye and 75 s left eye (normal 10–12 s) at baseline to normal single digit values over the 22-month period. Baseline Snellen visual acuity was 20/25 R, 20/20 L and remained at this level at the second visit. By the third visit at five months, the visual acuity right eye improved to 20/20 and remained unchanged by 22 months.

Case 3: A 67-year-old with Polypoidal Choroidal Vasculopathy (PCV), a “treatment resistant” AMD variant who has been on L/RV for almost 2 years. Three measures of ocular structure were enhanced. Retinal autofluorescence diminished (Figure 9), choroid thickened (Figure 10), and macular pigment volume improved (Figure 11). Two measures of visual function, CSF and GR, normalized (Figure 12). Significantly, all five measures were more notable in his more involved right retina.

## 4. Discussion

Diminishment of lipofuscin autofluorescence, as seen in Case 1 and 2, is pathognomonic of healthy retina-RPE-choroidal tissue, as is denser macular pigment (Case 1 and 3). The choroid typically thins with age and at a faster rate in AMD [23,24], yet in our patients, thickening was observed (Cases 1–3). Taken together, these clinical observations are consistent with the murine cardiac reperfusion experiments performed under the direction of Professor Depak Das at the University of Connecticut’s Cardiovascular Research Center [25,26,27], as well as a human clinical trial from Japan demonstrating superior vascular health in patients taking L/RV even for short periods of time [20]. Furthermore, the improved contrast sensitivity and glare recovery we observe are consistent with these improved structural observations.

Foveal health is ultimately affected by drusen deposits that degrade the function and number of cone photoreceptors. Biochemical signs of photoreceptor degeneration, such as decreased expression of synapse-associated proteins and increased expression of stress-response molecules, are seen in AMD patients. The salutary broad and comprehensive effects of L/RV on choroidal thickness, anti-inflammatory markers, regulation of divalent metals, the vitamin D pathway, cellular stress, toxicity genes, up-regulation of Nrf2, and even macular pigment volume appears to ultimately retard degeneration of photoreceptors.

Epigenetics is at the epicenter of modern medicine. It is the study of non-DNA sequence-related heredity that helps explain the relationship between an individual’s genetic background, the environment, aging, and disease. In 2013, researchers from a multinational consortium reported results on 19 common susceptibility loci from DNA analyses on nearly 80,000 people, and in aggregate, these loci explain only 10%–30% of the phenotypic variation of AMD [28,29]. The epigenetic state varies among tissues during a lifetime, whereas the DNA sequence remains essentially the same. Dietary recommendations or lack thereof, changes ones DNA and its translation. Clinical reports from our medical center provides us reason to believe that L/RV is beneficial because it reestablishes retinal architecture, decreases lipofuscin accumulation, increases choroidal thickness (perfusion), increases macular pigment volume, improves contrast sensitivity, decreases glare recovery time, and improves visual function—all ostensibly through improved retinal-RPE-choroidal cellular health. These observed clinical benefits in ocular structure and function are seen bilaterally in both eyes with further synergistic effects observed in cardiovascular parameters (*i*.*e*., Case 1), consistent with animal data, and the only human clinical trial to date [20,23,24,25].

## 5. Conclusions

Randomized placebo controlled ocular-cardiovascular molecular medicine clinical studies are warranted to confirm the proposed dual cardiovascular and ophthalmologic benefits of low-dose epigenetic nutriceutical intervention beyond AREDS II supplementation.
